# MicroRNA 320, an Anti-Oncogene Target miRNA for Cancer Therapy

**DOI:** 10.3390/biomedicines9060591

**Published:** 2021-05-23

**Authors:** Yuanyuan Liang, Shun Li, Liling Tang

**Affiliations:** 1 Key Laboratory of Biorheological Science and Technology, Ministry of Education, College of Bioengineering, Chongqing University, Chongqing 400044, China; 201919021022@cqu.edu.cn; 2 Department of Immunology, School of Basic Medical Sciences, Chengdu Medical College, Chengdu 610500, China; 3 Non-Coding RNA and Drug Discovery Key Laboratory of Sichuan Province, Chengdu Medical College, Chengdu 610500, China

**Keywords:** miR-320, EMT, tumor-suppressive, biomarker, therapeutic sensitivity

## Abstract

MicroRNAs are a set of highly conserved non-coding RNAs that control gene expression at the post-transcriptional/translational levels by binding to the 3′-UTR of diverse target genes. Increasing evidence indicates that miRNAs not only play a vital role in many biological processes, but they are also frequently deregulated in pathological conditions, including cancer. The miR-320 family is one of many tumor suppressor families and is composed of five members, which has been demonstrated to be related to the repression of epithelial-mesenchymal transition (EMT) inhibition, cell proliferation, and apoptosis. Moreover, this family has been shown to regulate drug resistance, and act as a potential biomarker for the diagnosis, prognosis, and prediction of cancer. In this review, we summarized recent research with reference to the tumor suppressor function of miR-320 and the regulation mechanisms of miR-320 expression. The collected evidence shown here supports that miR-320 may act as a novel biomarker for cancer prognosis and therapeutic response to cancer treatment.

## 1. Introduction

MicroRNAs (miRNAs or miRs) are short, single-stranded non-coding RNA molecules with a length of about 22 nucleotides that can directly combine with the 3′-UTR of their target mRNAs to induce their degradation or inhibit their translation [1]. Some studies have shown that miRNAs can also play a regulatory role on gene transcription by binding to the 5′UTR of a target gene [2,3,4,5,6,7,8]. Their roles in cancer may also be related to tumor inhibition or carcinogenesis. It has been reported that miRNAs control the expression of about 30% of human genes, making them vital for normal physiological conditions and development [5]. Thus, miRNAs have been linked to various signal pathways through the regulation of these target genes, which in turn regulates crucial biological processes that are often recognized as hallmarks of cancer, such as proliferation, invasion, metastasis, apoptosis, autophagy, and differentiation [6,7,9,10,11,12]. Additionally, miRNAs have been found to be dysregulated in many disease models, such as cardiovascular diseases and neurodegenerative diseases, as well as leukemia, and particularly in cancer [13,14,15,16]. Overall, the functions of miRNA vary greatly, and are different depending on the specific miRNA’s pathological type and physiological environment, which can make them carcinogenic factors to promote tumor progression, or as a tumor suppressors, to inhibit tumor cell metastasis.

The miRNA-320 family is comprised of miR-320a (chr8), miR-320b (chr1), miR-320d (chr13 and chrX), miR-320c (chr18), and miR-320e (chr19). The miRNA-320 family is closely related to human health and disease [17]. miR-320 itself is directly encoded by the upstream region of the cell cycle gene *RNA polymerase III subunit D (POLR3D)*, which is a specific conserved subunit of RNA polymerase III [18,19].

Previous reports have shown that the miR-320 family is lowly expressed in the ischemic heart, and protects against I/R-induced cardiomyocyte death and apoptosis [20]. The dysregulation of miR-320 in several cancers has attracted increasing interest as of late. Many studies have confirmed that miR-320 is downregulated during tumorigenesis and serves as a crucial suppressor of EMT, tumor proliferation, and metastasis [21,22,23,24]. In this review, we mainly focused on the latest findings regarding miR-320 in the study of tumor inhibition. Specifically, we included observations on the role of miR-320 in regulating EMT, cell proliferation, and apoptosis, alleviating drug resistance, and functioning as a biomarker for the diagnosis, prognosis, and prediction of cancer. Additionally, we further summarize the mechanism of miR-320 expression regulation in different cancer cells. All relevant studies are of great significance for understanding the anti-cancer signatures of miR-320. We sincerely hope that this review can provide useful insights into the use of miR-320 as a potential biomarker in the clinical setting and as a therapeutic target in cancer treatment.

## 2. Tumor Suppressive Functions of miR-320

### 2.1. miR-320 Inhibits EMT and Tumor Metastasis

A number of studies have indicated that the expression of miR-320 family members was negatively associated with the EMT-related phenotype of cancer cells, as demonstrated by N-cadherin-negative and E-cadherin-positive cancer cell lines. An increasing number of findings have shown that miR-320 family members were negatively regulated in tumor cell migration and invasion [24], which suggested that miR-320 was associated with the EMT process. Hong et al. [21] found that hsa-miR-320a-3p was involved in the regulation of EMT, functioning by suppressing the level of *E-cadherin* and upregulating expression of *N-cadherin* and vimentin by directly targeting *forkhead box M1 (FOXM1).* In general, there are two ways that *miR-320* negatively controls EMT to play its tumor-suppressive role.

First, miR-320 can regulate EMT through binding with the 3′-UTR of EMT-related transcription factors (EMT-TFs), which are indispensable for the process of EMT. Of these EMT-TFs, *twist family bHLH transcription factor 1 (TWIST1)* can regulate the expression of E-cadherin via inducing promoter hypermethylation and chromatin modification of target genes. It has been reported that a hypermethylated promoter for *TWIST1* was related to several types of cancers [25]. Moreover, the authors also found that the metastasis rate of hypermethylated *TWIST1* was higher in primary breast carcinomas, indicating that *TWIST1* may be involved in EMT regulation through promoter hypermethylation [26]. However, the latent mechanism of action for *TWIST1* remains unclear. Additionally, *TWIST1* suppresses E-cadherin expression via activating the transcription of *BMI1 proto-oncogene, polycomb ring finger (BMI1)*, a chromatin modifier, indicating that the chromatin modification function of *TWIST1* could be related to EMT [27]. Dual-reporter assay findings indicated that *TWIST1* could directly regulate miR-320 expression [28]. It was shown that there is a conserved miR-320 seed sequence in the *TWIST1* 3′UTR and miR-320 can modulate the activity of *TWIST1*. Moreover, the *BMI1* 3′-UTR also has a conserved sequence that can pair to hsa-miR-320a-3p, suggesting that *TWIST1* may indirectly regulate miR-320 expression via interaction with *BMI 1* [29]. Therefore, a *TWIST1/*miR-320 feedback loop could control the initiation of EMT. The research mentioned above shows that miR-320 family members inhibit EMT-related TFs, resulting in EMT attenuation.

**Figure 1:**
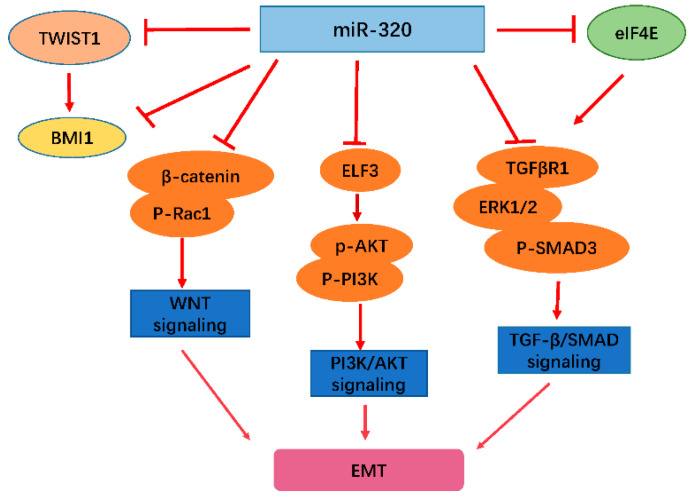
Schematic of the mechanism of miR-320 regulation of EMT. miR-320 inhibits EMT by interacting with TWIST1 and EMT-associated signal pathways. For example, miR-320a suppresses the expression of β-catenin and p-Rac1 to regulate the WNT and PI3K/AKT pathways, which are also repressed through decreasing p-AKT and p-PI3K. miR-320 can also attenuate the activity of TGF-β/SMAD by suppressing TGFβR1, EGK1/2, and p-SMAD3 expression. Through the above important signal pathways, miR-320 achieves the modulation of EMT. BMI1: polycomb ring finger, ELF3: E74 like ETS transcription factor 3, elF4E: eukaryotic translation initiation factor 4E, Rac1: Rac family small GTPase 1, and TWIST1: twist family bHLH transcription factor 1.

Second, the miR-320 family regulates EMT through some important signal pathways, for example, the Wnt, PI3K/Akt, and TGF-β/Smad signaling pathways [30]. miR-320 can negatively control TGF-β/Smad signaling by regulating target gene expression [30,31,32]. It has been reported that miR-320a, miR-320b, and miR-320d suppress TGFβ1-induced EMT through targeting the 3′ UTR of the TGFβR1 transcript [30]. Moreover, hsa-miR-320a-3p was shown to prevent TGFβ1-induced EMT by targeting *p-eIF4E* and inhibiting the migration and invasion of endometrial cancer cells [31]. In addition, hsa-miR-320a-3p was found to suppress EMT progress and affect the protein expression of *cyclin dependent kinase 2 (CDK2)* and *matrix metallopeptidase 2 (MMP2)* by reducing the phosphorylation levels of ERK1/2 [32]. miR-320 can also negatively control Wnt signaling by regulating its pathway-associated genes [33,34,35,36]. As a downstream component of the Wnt signaling pathway, *FOXM1* binds directly to β-catenin, and thus enhances the nuclear localization of *β-catenin* and its transcriptional activity [33]. It has also been reported that miR-320 can decrease *FOXM1* expression via directly binding to its 3′UTR, resulting in the repression of migration and invasion of colorectal cancer (CRC) cells [34]. Furthermore, Heieh et al. [35] found that *β-catenin* expression was decreased by hsa-miR-320a-3p at the posttranscriptional level via directly targeting a specific site within the 3′-UTR of *β-catenin*, which led to the inhibition of the development and progression of prostate cancer cells. Moreover, *ΔNp63α*, a member of the p53 family of TFs, inhibits *Rac family small GTPase 1 (Rac1)* phosphorylation via positively regulating hsa-miR-320a-3p expression, which results in a reduction in noncanonical WNT signaling and EMT [36]. As is known to all, activation of PI3K/Akt signaling is involved in different cellular processes and promotes the progression of several cancers [37,38,39]. A previous study showed that hsa-miR-320a-3p suppressed cell migration and invasion via inhibiting PI3K/Akt signaling in non-small cell lung cancer (NSCLC) cells [37]. Moreover, Zhang et al. [38] found that miR-320 regulates EMT and the PI3K/AKT signaling pathway by targeting ELF3 in breast cancer. Moreover, ELF3 partially rescued the miR-320-mediated inhibitory effect on the PI3K/AKT signaling pathway. The expression of these miR-320 family members has been shown to be dysregulated in various human cancer cells, which leads to EMT and tumor progression. Thus, restoring miR-320 family member expression attenuates EMT in CRC, NSCLC, melanoma, endometrial cancer, hepatocellular carcinoma (HCC), osteosarcoma, prostate cancer (PCa), and nasopharyngeal carcinomas (NPC) [28,40,41,42,43,44,45,46]. Furthermore, it is worth noting that miR-320 regulation might be related to low survival rate, and can be employed as a prognostic marker for cancer patients [24,47,48,49,50,51,52,53]. Thus, findings related to miR-320 and EMT will lay a foundation for future studies on the anti-tumor functions of miR-320 (Figure 1).

miR-320 has been confirmed as a microRNA correlated with metastasis and invasion, particularly in colorectal cancer, glioblastoma, NSCLC, and breast cancer (BC) cell lines by targeting crucial genes to decrease metastatic potential, such as *G protein subunit alpha i1 (GNAI1)*, *early growth response 3 (EGR3)*, and *E74 like ETS transcription factor 3 (ELF3)* [37,54,55]. A recent study reported conflicting evidence that ectopically-expressed miR-320a/c/d significantly enhanced the migration and invasion of SK-Hep—1 cells by targeting *GNAI 1*, which is a member of the Gα inhibitory family [54]. Tang et al. [55] demonstrated that hsa-miR-320b could directly target *EGR3*, a zinc-finger transcription factor, repressing invasion and metastasis in glioblastoma cells. They also found that overexpression of hsa-miR-320b by binding to *EGR3* inhibited the expression of p-PI3K and p-Akt, suggesting that hsa-miR-320b was related to the EGR3-induced modulation of glioblastoma cell malignant progression that occurs via the EGFR/PKP2 pathway. Through studying different glioma cell lines with distinct capabilities to metastasize, it was shown that increasing the expression of hsa-miR-320b could inhibit glioma cell migration and invasion by significantly down regulating the expression of *matrix metallopeptidase 2 (MMP2)* and *matrix metallopeptidase 9 (MMP9)* [24]. A recent study demonstrated inverse results when compared with related CRC tissues, where hsa-miR-320b was upregulated in colorectal cancer patients with liver metastasis [56]. The authors found that enforced expression of hsa-miR-320b could upregulate the target genes of hsa-miR-320a-3p including *β-catenin*, *Neuropilin-1*, and *Rac-1*, which were all demonstrated to promote tumor proliferation, invasion, and metastasis. It was noticed that, in this research, hsa-miR-320b and hsa-miR-320a-3p had different target genes and seed sequences, which may have resulted in differences in the modulation of tumor metastasis. In addition, Zhao et al. [37] found that overexpression of has-miR-320a-3p suppressed cell migration and invasion through the inhibition of the PI3K/AKT pathway in NSCLC cells. According to other recent studies supporting the notion that miR-320 functions as a tumor repressor, other evidence is needed for demonstrating that miR-320 plays a carcinogenic role. Several studies have shown that miR-320 is upregulated in tumors and promotes proliferation, migration, invasion, and reduced cancer cell sensitivity to chemotherapeutics [54,57,58], suggesting that miR-320 pro-tumoral or anti-tumoral functions are context-dependent. Combined with the above studies from various cancers listed in Table 1, many figures have indicated that the miR-320 family plays a vital role not only in repressing EMT, but also in tumor cell metastases and invasion. Therefore, miR-320 will hopefully become a novel tumor prognosis biomarker and a target for new medicine development to suppress disease progression.

### 2.2. The Modulatory Role of miR-320 in Cell Proliferation and Apoptosis

Many studies have shown that the miR-320 family was involved in modulating the proliferation of cancer cells. Wang et al. [22] was the first study to describe that miR-320 could regulate cell growth in bladder cancer. They also found that overexpression of *hsa-*miR-320c could trigger G1-phase arrest, which may have been due to decreased expression of *cyclin dependent kinase 6 (CDK6)* [22]. In lung adenocarcinoma cells, has-miR-320a can markedly inhibit tumor cell growth through suppressing *signal transducer and activator of transcription 3 (STAT3)* expression and its downstream signals, which was linked to the inhibition of cell growth in a xenograft mouse model of lung adenocarcinoma [23]. In addition, Tadano et al. [79] found that overexpression of hsa-miR-320e suppressed tumor proliferation, which may have been linked to decreased expression of *CDK6*. More recently, Wu et al. [80] demonstrated that forced expression of miR-320a/b/c/d/e inhibited cell proliferation and resulted in cell cycle arrest in osteosarcoma cells by targeting *E2F transcription factor 1 (E2F1)*, a cell cycle regulator. In addition, Lv et al. [24] found that enforced expression of has-miR-320b resulted in a G0/G1 phase arrest through dramatically reducing the expression of *Cyclin D1* and *BCL2 apoptosis regulator (BCL-2*), and increasing the *BCL2 apoptosis regulator (Bax)* level. Therefore, these studies support the notion that miR-320 can play a crucial role in diverse types of cancers, including bladder cancer, lung cancer, and osteosarcoma.

**Figure 2:**
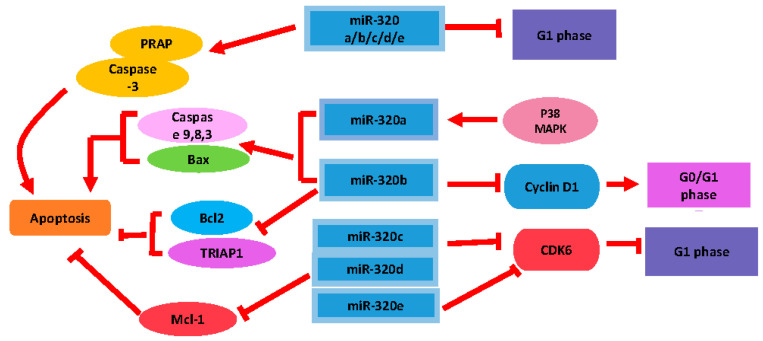
miR-320 is involved in the regulation of cell proliferation and apoptosis. Bax: BCL2 apoptosis regulator, BCL-2: BCL2 apoptosis regulator, CDK6: cyclin dependent kinase 6, Mcl-1: MCL1 apoptosis regulator, PRAP: proline rich acidic protein, and TRIAP1: TP53 regulated inhibitor of apoptosis 1.

Apart from taking part in cell cycle regulation, the miR-320 family has been shown to regulate apoptosis. In multiple myeloma, apoptosis was modulated by the miR-320 targeting of *Pre-B-cell leukemia transcription factor 3 (PBX3)* [81]. Furthermore, the expression of some apoptosis-related proteins was measured and found to be decreased in this study, such as *proline rich acidic protein 1 (PRAP1)* and *caspase-3*. Lin et al. found that hsa-miR-320d could directly downregulate *MCL1 apoptosis regulator (Mcl-1)* protein expression, thereby promoting PCa cells apoptosis [82]. In addition, hsa-miR-320b was shown to restrain NPC cell proliferation and enhance mitochondrial fragmentation and apoptosis both in vitro and in vivo by targeting *TP53 regulated inhibitor of apoptosis 1 (TRIAP1),* which is a TP53-regulated inhibitor of apoptosis [83]. Moreover, it was also found that hsa-miR-320a-3p, by inhibiting the expression of both STAT3 and phosphorylated *p-STAT3 (p-STAT3)*, further induced lung cancer cell apoptosis [23]. In a previous study, *the p38 MAPK* and *JNK* pathways were able to induce the expression of hsa-miR-320a-3p, and this finding was closely implicated in the suppression of apoptosis in Hela cells [84]. In addition, the targeting effect of hsa-miR-320a-3p on *X-linked inhibitor of apoptosis (XIAP)*, a member of the inhibitors of apoptosis (IAP) family, was also confirmed. To sum up, these studies indicated that miR-320 can control apoptotic processes via some of its targets and via inducing their inhibition (Figure 2).

### 2.3. The Modulatory Role of miR-320 in Alleviating Drug Resistance

The downregulation of miR-320 has been reported to be relevant to drug resistance to treatments such as oxaliplatin, epirubicin, gemcitabine, tamoxifen, and doxorubicin [34,77,85,86,87,88,89]. However, the drug resistance mechanisms utilized in these examples are not fully understood. Interestingly, some reports have found that the recovery of miR-320 expression can effectively alleviate drug resistance to certain drugs [34,77,88]. For example, Wan et al. [34,63] reported that overexpression of miR-320 could elevate the sensitivity of CRC cells to 5-Fu and oxaliplatin by targeting *forkhead box M1 (FOXM1)*. Chong et al. reported that upregulation of hsa-miR-320a-3p decreased the sensitivity of primary BC cells to epirubicin [85]. Moreover, the expression of hsa-miR-320a-3p was significantly negatively associated with anthracycline sensitivity. Lim et al. [77] reported that recovery of hsa-miR-320c expression could increase the responsiveness to oxaliplatin in triple-negative breast cancer (TNBC) through targeting *checkpoint kinase 1 (Chk1),* which regulated DNA damage responses. When examining the miRNA expression of cells treated with gemcitabine-based chemotherapy, maladjustment of hsa-miR-320c was associated with an attenuated sensitivity to gemcitabine in pancreatic cancer [86]. In ionizing radiation (IR)-treated HCC tissues, hsa-miR-320b was specifically downregulated [87]. The authors found that suppression of *RAD21 cohesin complex component (RAD21)*, a DNA repair gene, significantly attenuated the effects of hsa-miR-320b inhibition on IR-induced DNA damage. Recently, Lu et al. [88] found that hsa-miR-320a-3p was downregulated in tamoxifen-resistant BC cells. However, the recovery of hsa-miR-320a-3p expression could recover tumor cell sensitivity to tamoxifen [88]. These mechanistic studies have indicated that this effect was mainly effected by targeting *cAMP-regulated phosphoprotein (ARPP-19)* and *estrogen-related receptor gamma (ERRγ)* and their downstream effectors, MYC binding protein *(c-Myc)* and *Cyclin D1*. Moreover, He et al. [89] also found that miR-320 played a vital role in chemoresistance by targeting *transient receptor potential channel C5 (TRPC5)* and *nuclear factor of activated T-cells isoform c3 (NFATC3)*. Zhou et al. found that hsa-miR-320a-3p was associated with doxorubicin resistance in osteosarcoma by targeting of *SNGN12* [90].

In short, apart from the well-known inhibitory functions on EMT, miR-320 has been confirmed to function through several novel tumor-suppressive pathways in several human cancers, such as through modulation of cell division and apoptosis and alleviation of drug resistance. These findings highlight the idea that miR-320 is a key molecular biomarker for oncogenesis and progression.

## 3. miR-320: A Biomarker for Diagnosis, Prognosis, and Prediction of Cancer

As is mentioned above, miR-320 is one of the tumor suppressors identified that is related to tumorigenesis and progression. Additionally, it has been found to be downregulated in several cancers. These findings suggest the importance of miR-320 as a valuable biomarker.

The use of microRNAs as biomarkers is increasing due to their stability in bodily fluids, which makes them easy to assay [24,48,91]. As previously mentioned, miR-320 is downregulated in many cancers, such as breast, lung, prostate, cervical, colon, gastric cancer, and glioma [21,24,36,43,53,87,92]. Furthermore, miR-320 targets vital oncogenes as well as suppressor genes, such as *E74 like ETS transcription factor 3* (*ELF3)*, *early growth response 3* (*EGR3)*, and *TRIAP*1 [37,55,83]. For this reason, many studies have proposed that miR-320 could be used as a potential biomarker for diagnosis, prognosis and cancer prediction, as well as a therapeutic target for drug development.

### 3.1. miR-320 as a Diagnositic Biomarker

miR-320 has demonstrated its potential as a diagnostic biomarker for several types of cancers such as colorectal, breast cancer, and retinoblastoma. miR-320 was shown to be downregulated in the serum of colorectal carcinoma patients, with a sensitivity of 0.93 and specificity of 0.73 [92]. These results were higher than those of other known markers, for example, CEA and CA19-9, which have a sensitivity of 10% and 15% in first stage CRC, respectively [93]. As it has correspondingly high sensitivity and specificity in contrast to other markers, miR-320 is recommended for further study to verify its clinical application for diagnosis.

As in colorectal cancer, miR-320 dysregulation can also be used as a diagnostic marker in breast cancer. Ozawa et al. [94] reported that miR-320 was abnormally downregulated in breast cancer samples (31 serum samples). In this study, the authors proposed a three-miRNA panel (hsa-miR-142-5p, has-miR-320a-3p, and hsa-miR-4433b-5p) for the non-invasive diagnosis of breast cancer. The authors of this study mentioned that the high sensitivity and specificity in breast cancer serum samples were 93.33% and 68.75%, respectively, and with an area under the curve (AUC) was 0.8387. Liu et al. found that the expression of miR-320 was reduced in retinoblastoma (RB) samples (65 patients) [95]. The authors proposed a novel panel that combined miR-320 with neuron-specific enolase (NSE), based on existing diagnostic methods. Moreover, the sensitivity and specificity of this combination was 0.98, and with an AUC of 0.996. The above results may provide a possibility for the early diagnosis of RB. However, further studies are required to assess the accuracy and reliability of miR-320 as a plasma biomarker of RB.

The downregulation of miR-320 in different types of cancers, such as breast, gastric, colorectal, and prostate cancers, makes it possible to use miR-320 as a non-invasive diagnostic biomarker. However, this should be confirmed more widely in other cancers. Thus, a wider range of validation studies a needed to further study before the clinical application of each cancer.

### 3.2. miR-320 as a Prognostic Biomarker

Many studies have indicated that miR-320 can be used as a prognostic biomarker in CRC, salivary adenoid cystic carcinoma (SACC) and glioma (GBM). Fang et al. found that the concentration of plasma miR-320 was reduced in 111 patients with CRC [95]. In this study, the patients with low plasma levels of miR-320 had a high hazard of liver metastasis six months after CRC operation. The above results suggested that the plasma level of *miR-320* had potential prognostic value after surgery [95]. In another study, Sun et al. [96] found that in SACC samples, miR-320 was significantly downregulated and closely associated with lung metastasis. This study demonstrated that low levels of miR-320 had a high cumulative metastasis rate and low survival rate, which were 52.4% and 44.4%, respectively. Interestingly, the authors found that miR-320 could predict lung metastasis in SACC (HR = 0.45), independent of the TNM stage. A recent study proposed that miR-320 expression was positively correlated with patient survival in glioma [71]. They found that higher expression of miR-320 correlated with longer disease-free survival (DFS) and overall survival (OS). The TCGA database was used to further verify the prognostic value of miR-320a in GBM [71]. These results demonstrated that miR-320 was an underlying prognostic biomarker for GBM patients.

Although miR-320 has been linked to the prognosis of several types of cancer, specific research and additional information is needed to confirm whether this is generally associated with each type of cancer.

### 3.3. miR-320 as a Predictive Biomarker

In addition to its use as a diagnostic and prognostic biomarker in a diverse range of cancers, miR-320 has also been shown to be a promising predictive biomarker in NSCLC, HCC, and CRC. This is mostly due to resistance to therapeutic drugs that some cancers develop. Decreased miR-320 levels were also shown to be linked to resistance to cisplatin-based chemotherapy in patients with NSCLC [97]. In this study, resistant responders had decreased levels of miR-320 compared with sensitive responders. Although the mechanism related to *miR-320* resistance is still unclear, it was also found that target genes of miR-320 were involved in tumor progression and cell cycle progression, as well as the MAPK and ErbB signaling pathways, which can promote chemoresistance to cisplatin [60,98].

Additionally, miR-320 was shown to play a pivotal role in sensitivity to IR treatment in patients with HCC [87]. A low level of tissue miR-320 was related to a poor response to IR treatment in 20 patients. The authors of this study linked the decreased sensitivity to IR to DNA damage resistance after treatment, as miR-320 targets. *RAD21 cohesin complex component (RAD21)*, a component of the cohesion complex that is crucial for DNA damage repair [99]. The above results suggested that miR-320 has a level of potential predictive value after surgery. In this condition, it was found that miR-320 levels were related to clinical and non-clinical chemotherapy resistance. However, these could not be distinguished between pathologic responses and non-pathologic responses in the same study. Thus, more validation and information on miR-320 as a predictive biomarker for chemotherapy is needed, and multicenter studies would be useful for this purpose.

## 4. Regulation of *miR-320* Expression

Transcriptional regulation and epigenetic modification are two significant mechanisms that control miRNA expression [57,64,100,101]. As the miR-320 family acts as a tumor repressor has attracted increasing interest in many human tumors, and transforming the tumor suppression signal from miR-320 may be a new strategy to treat cancers in the future. Here, we evaluated recent findings for miR-320 expression modulation.

### 4.1. Regulation of Transcription Factors

The regulation mechanisms for miR-320 expression have been studied in several cell types, and some pivotal TFs were demonstrated to be relevant to this process. A recent study reported that *lncRNA TTN-AS1* was able to downregulate hsa-miR-320a-3p through post-transcriptional regulation in cholangiocarcinoma cells, which depended on the Ago2 pathway [64]. *E2A* can realize self-regulation as a transcription factor in the process of accelerating the disease progression of CRC cells [101]. *E2A* was found to directly bind to miR-320a’s E-box, thereby upregulating its expression [101]. Costa et al. [57] found that the inhibitory function of *p53* on tumor escape was involved the transactivation of hsa-miR-320a-3p. *hsa-miR-320a-3p* repression of *PDL1* was associated with *p53* regulated tumor escape [57]. In addition, Δ*Np63α* is a member of the *p53* TF family, which regulates the transcription of *miR-320* by binding to the *p63* binding site 6 kb downstream of *hsa-miR-320a-3p* [37]. Hu et al. [84] found that *transcription factor 2 (AFT2)*, *ETS transcription factor (ELK1),* and *YY1 transcription factor (YY1)* could directly bind the hsa-miR-320a-3p promoter and activate hsa-miR-320a-3p expression under IR conditions. A previous study reported that the transcription factor *cAMP responsive element binding protein 1 (CREB1)* could bind to the promoter of hsa-miR-320-3p to induce its expression, which facilitated mitophagy in cervical cancer cells [100]. In laryngeal carcinoma cells, *AFAP1-AS1* can upregulate *recombination signal binding protein for immunoglobulin kappa J region (RBPJ)* expression by negative regulation of hsa-miR-320a-3p, and enforced expression of *RBPJ* could reduce the stemness and chemoresistance inhibited by *AFAP1-AS1* silencing [73].

As discussed above, many different molecules have been shown to regulate miR-320 expression, which leads to diverse downstream effects on cancer.

### 4.2. Epigenetic Modification

In addition to the regulatory expression supported by the aforementioned TFs, epigenetic modification was identified to regulate miR-320 family expression in cancer cells, for example, by regulation of DNA methylation. DNA methylation usually occurs at CpG islands in gene promoter regions, and has been shown to be related to gene silencing [102]. Several studies have indicated that the regulatory regions of *miR-320* include abundant CpG sequences [89]. He et al. [89] found that highly methylated CpG islands were present in miR-320 low level chemoresistant tumor cells. However, in cells with high expression of hsa-miR-320a-3p, their CpG islands are unmethylated, which indicates that DNA methylation regulates the expression of hsa-miR-320a-3p in tumor cells. Li et al. [103] reported that hsa-miR-320a-3p was hypermethylated and downregulated in GC tissues and cell lines. Roy et al. [104] found that hsa-miR-320b was silenced at the same time as DNA hypermethylation in oral cancer, and was associated with worse five-year survival. Thus, these above results indicated that miR-320 expression regulated by DNA methylation seemed to relate to chemotherapy resistance and increased tumor progression.

**Figure 3:**
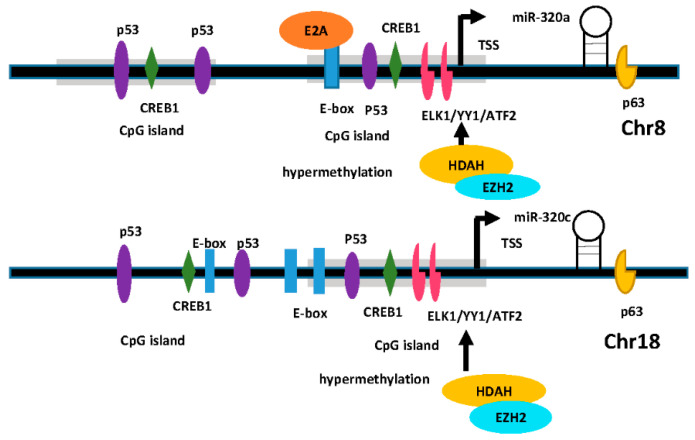
Regulation of miR-320 expression involving multiple transcription factors (TFs) and epigenetic modification. TFs and epigenetic modification are involved in regulation of miR-320a and *miR-320c*. For example, *E2A* can directly bind to miR-320a’s E-box and upregulate its expression. Δ*Np63α* regulates the transcription of miR-320 by binding to the *p63* binding site in 6 KB region downstream of miR-320a-3p. Moreover, *AFT2*, *ELK1,* and *YY1* can directly bind the miR-320a/c promoter and induce miR-320a/c expression. *Histone methyltransferase enhancer of zeste homolog 2 (EZH2)* inhibition results in the reactivation of the expression of hsa-miR-320a/c. TSS represents transcriptional starting site.

DNA methylation and histone modification are another epigenetic pattern that can modulate the expression of miR-320 family members. Sato et al. [76] found that in three PCa cell lines, the expression of hsa-miR-320a-3p was increased after treatment with OBP-801, a histone deacetylase (HDAC) inhibitor. The epigenetic results show that the acetylation levels of H3K9 in the hsa-miR-320a-3p promoter in PC cell lines were notably increased after OBP-801 treatment [76]. Furthermore, hsa-miR-320c was also significantly increased by OBP-801 treatment. Alzrigat et al. [105] found that treatment of multiple myeloma (MM) patients with a *histone methyltransferase enhancer of zeste homolog 2 (EZH2)* inhibitor resulted in the reactivation of the expression of hsa-miR-320c, leading to downregulated expression of MM-associated oncogenes. Sun et al. [106] found that hsa-miR-320a-3p was silenced primarily through histone methylation in human fetal ovarian cells. Therefore, it is legible that epigenetic modulation plays a vital role in the regulatory expression of the miR-320 family (Figure 3).

## 5. Conclusions and Perspectives

The miR-320 family, as a negative regulator of tumor related-EMT, plays a pivotal role in suppressing oncogenesis. Given that the miR-320 family serves as a negative regulatory factor for tumor-related EMT to inhibit the progression of malignant tumors, future work may enhance our understanding of the effects of miR-320 on tumorigenesis through identifying additional downstream targets and realizing a functional characterization of *miR-320*. Additionally, miR-320 functions in tumor inhibitory effects, such as the inhibition of cell proliferation and apoptosis, a reversal of chemoresistance and as a potential prognosis biomarker. Hence, miR-320 may be a novel therapeutic target for cancer based on these important studies. In addition to reviewing the latest progress of miR-320 in tumor suppressive functional, we also collated the findings of recent research and summed up the regulatory mechanisms acting on miR-320 expression. Due to the non-coding nature of miR-320, multiple TFs involved in transcription regulation and epigenetic modulation are the main regulatory effectors of miR-320. Understanding the above regulatory mechanisms can not only enhance our outstanding of the tumor inhibitory effect of miR-320, but also allow us to embrace miR-320 to develop new drugs for these small molecules.

In recent years, there are an increasing number of studies on miR-320 family member and cancer, which demonstrates the increasing interest in the research of miR-320. Due to the tumor suppressive properties of miR-320, it is possible that restoring expression of miR-320 can be deemed as a novel therapy for human cancer treatment. However, the mechanistic basis of miR-320 anticancer activity still needs to be further studied. miR-320 plays a crucial role in relieving drug resistance, which revels that miR-320 may be a potential new drug resistance target. miR-320, as both a prognostic biomarker and a novel therapeutic target, could further improve the efficacy of radiotherapy and chemotherapy. However, most of the miR-320 activities described display a limited clinical relevance as prediction markers because their potential role comes from in vitro observations in cell lines, and no clinical data are currently available from patients who have undergone external treatment. At present, an effective delivery vector yields the possibility for miR-320 to overcome numerous extracellular and intracellular obstacles. Unfortunately, no studies have shown a suitable miR-320 delivery vector for cancer treatment. Therefore, further investigation is required, and this review may offer new insights for the diagnosis of early cancers, screening of patient treatment response, and the prediction of prognosis.

## Figures and Tables

**Table 1:** Dysregulation of *miR-320* in various human cancers.

Tumor Types	*miR-320* Family Member	Number of Tumor Types	Expression Level Compared with Normal Tissues	Ref.
BC	miR-320a	19	Down	[59]
BC	miR-320a	30	Down	[60]
BC	miR-320a	36	Down	[61]
Bladder cancer	miR-320a	65	Down	[62]
Bladder cancer	miR-320c	13	Down	[22]
CCA	miR-320a	27	Down	[63]
CCA	miR-320a	39	Down	[64]
Cervical cancer	miR-320a	48	Down	[21]
Colon cancer	miR-320a	40	Down	[65]
CRC	miR-320a	62	Down	[66]
CRC	miR-320b	26	Down	[67]
Gastric cancer	miR-320a	22	Down	[68]
Gastric cancer	miR-320a	40	Down	[69]
GCA	miR-320d	60	Down	[53]
Glioma	miR-320a	42	Down	[70]
Glioma	miR-320a	120	Down	[71]
HCC	miR-320a/c/d	50	Up	[54]
HCC	miR-320a	42	Down	[72]
LAC	miR-320a	18	Down	[23]
MPM	miR-320a	14	Up	[57]
NPC	miR-320a	24	Down	[73]
NPC	miR-320a	68	Down	[74]
NSCLC	miR-320a-3p	80	Down	[37]
OSC	miR-320a	25	Down	[75]
PCa	miR-320a	51	Up	[58]
PCa	miR-320a	10	Down	[76]
TNBC	miR-320c	97	Down	[77]
TSCC	miR-320a	5	Down	[78]

BC: breast cancer, CCA: cholangiocarcinoma, CRC: colorectal cancer, GCA: gastric cardiac adenocarcinoma, HCC: hepatocellular carcinoma, LAC: lung adenocarcinoma, MPM: Malignant pleural mesothelioma, NPC: nasopharyngeal carcinoma, NSCLC: non-small cell lung cancer, OC: ovarian cancer, OSC: osteosarcoma, PCa: prostate cancer, TNBC: triple-negative breast cancer, and TSCC: tongue squamous cell carcinoma.
